# Intracerebral haemorrhage in Down syndrome: protected or predisposed?

**DOI:** 10.12688/f1000research.7819.1

**Published:** 2016-05-12

**Authors:** Lewis Buss, Elizabeth Fisher, John Hardy, Dean Nizetic, Jurgen Groet, Laura Pulford, André Strydom

**Affiliations:** 1 Division of Psychiatry, University College London, London, UK; 2 Institute of Neurology, University College London, London, UK; 3 Lee Kong Chian School of Medicine, Nanyang Technological University, Singapore, Singapore; 4 Blizard Institute, Barts and the London School of Medicine, Queen Mary, University of London, London, UK; 5 London Down Syndrome (LonDownS) Consortium, University College London, London, UK

**Keywords:** Intracerebral haemorrhage, Down syndrome, trisomy, Alzheimer’s disease, Cerebral amyloid angiopathy

## Abstract

Down syndrome (DS), which arises from trisomy of chromosome 21, is associated with deposition of large amounts of amyloid within the central nervous system. Amyloid accumulates in two compartments: as plaques within the brain parenchyma and in vessel walls of the cerebral microvasculature. The parenchymal plaque amyloid is thought to result in an early onset

Alzheimer’s disease (AD) dementia, a phenomenon so common amongst people with DS that it could be considered a defining feature of the condition. The amyloid precursor protein ( *APP*) gene lies on chromosome 21 and its presence in three copies in DS is thought to largely drive the early onset AD. In contrast, intracerebral haemorrhage (ICH), the main clinical consequence of vascular amyloidosis, is a more poorly defined feature of DS. We review recent epidemiological data on stroke (including haemorrhagic stroke) in order to make comparisons with a rare form of familial AD due to duplication (i.e. having three copies) of the *APP* region on chromosome 21, here called ‘dup-APP’, which is associated with more frequent and severe ICH. We conclude that although people with DS are at increased risk of ICH, this is less common than in dup-APP, suggesting the presence of mechanisms that act protectively. We review these mechanisms and consider comparative research into DS and dup-APP that may yield further pathophysiological insight.

## Introduction

Down syndrome (DS), which is due to an extra copy of chromosome 21, is strongly associated with early onset Alzheimer’s disease (AD) ^1^. This is likely due to the presence of three copies of the gene coding for the amyloid precursor protein ( *APP*) situated on chromosome 21, resulting in typical pathological features of AD, including senile plaques, composed of small, insoluble fragments of APP referred to as amyloid β (Aβ), formed after cleavage by specific secretase enzymes. The ensuing neurocognitive decline is a striking clinical feature of DS.

Cerebral amyloid angiopathy (CAA) results from the deposition of amyloid within the walls of leptomeningeal and cerebral blood vessels ^2^ and is present in more than 80% of AD brains at post mortem ^3^. As in AD, this amyloid derives from APP and is also composed of Aβ fragments. The process of vascular amyloid deposition is largely silent; however, when severe, it may set off a cascade of events resulting in intracerebral haemorrhage (ICH), the main clinical consequence of CAA. CAA-related haemorrhages tend to affect the elderly and occur multiply and in cortical and subcortical (lobar) regions ^4^. Like AD, CAA occurs frequently in DS ^5^; however, unlike AD dementia, CAA-ICH is not a well-established clinical phenomenon in people with DS.

If CAA-ICH is over-represented in people with DS compared with the euploid population, it is important as an avenue for research and also to clinicians to provide more appropriate care to this group. In this review, we consider the rates of CAA and ICH in individuals with DS and compare these with sporadic AD as well as a specific form of familial AD due to duplication of the APP region on chromosome 21. Finally, we consider potential mechanisms for apparent differences between these groups.

## Intracerebral haemorrhage in Down syndrome: epidemiology

Until recently, data on CAA-ICH in DS have been limited. There are seven case reports of people with DS suffering severe ICH ^6– 11^. Their paucity and noteworthiness suggest that ICH is not part of the experience of clinicians caring for people with DS. However, several mortality studies have reported increased incidence of cerebrovascular events in people with DS ^12, 13^ but failed to distinguish between ischaemic and haemorrhagic stroke types.

More recently, Sobey *et al.* reported population-level data on cardiovascular events in 4081 people with DS and 16,324 age-matched controls ^14^. Both ischaemic (risk ratio [RR] = 3.76, 95% confidence interval [CI] 2.39, 5.92) and haemorrhagic (RR = 3.31, 95% CI 1.95, 5.60) strokes are reported as more common amongst people with DS than non-DS controls. The incidence rates for ‘any stroke’ were 1.3% in males and 2.3% in women aged 19 to 50. For those over 51 years, the corresponding values were 11.3% in males and 8.2% in women. For haemorrhagic stroke, the values were 3.8% in males and 3.3% in women older than 51. However, when corrected for existing cardiovascular risk factors (including hypertension, diabetes, smoking, cardiac arrhythmia, sleep apnoea, congenital heart disease, pulmonary hypertension, and Moyamoya disease), the increased risk is largely attenuated for ischaemic but not for haemorrhagic stroke. The authors propose that this excess risk may be accounted for by factors not adjusted for in the regression analysis, such as misclassification of ischaemic-haemorrhagic transformation or anticoagulation, but it is also possible that some of this increased risk for haemorrhagic stroke may be explained by the deposition of vascular amyloid seen in DS, supporting the view that CAA-ICH is relatively common in DS.

## Amyloid precursor protein duplication

The genetic abnormality in DS thought to underpin AD and CAA is the triplication of the *APP* gene, along with the rest of chromosome 21. It is proposed that this increased ‘dose’ of APP provides extra substrate for Aβ production, which then is deposited as senile plaques in AD or vascular amyloid in CAA.

APP is cleaved to Aβ fragments by γ-secretase enzymes whose catalytic subunit is coded for by *PSEN1* and *PSEN2* genes. There are many well-documented familial forms of AD caused by missense mutations in *PSEN1*, *PSEN2*, and the *APP* gene ^15^ that modulate APP processing and increase Aβ deposition. Similarly, hereditary forms of CAA, such as the Dutch type CAA, result from missense mutations in the same three genes ^16^.

In addition, a novel genetic form of AD has been recognised in the last 10 years arising from small internal chromosome 21 duplications ^17– 25^. These rare copy number variants all result in three copies of *APP*, collectively known as duplication of *APP* (dup- *APP*) ^26^, and lead to an APP overdose. In this sense, dup-APP differs from other forms of familial AD that are the result of point mutations in *APP*, *PSEN1*, or *PSEN2.* Meaningful comparison can be made with DS, as an additional copy of *APP* is present in both diseases; DS differs from dup-APP only in the number of other genes on chromosome 21 that are also trisomic.

**Figure 1.**
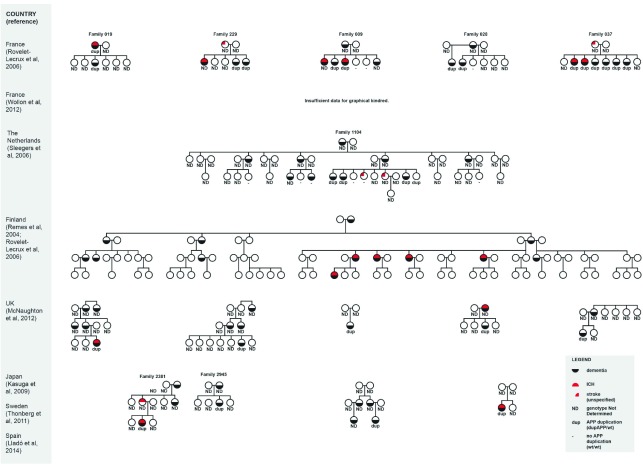
Summary of kindreds harbouring duplication (i.e. having three copies) of the amyloid precursor protein region on chromosome 21 (dup-APP). Phenotype with respect to dementia (black crescent) and intracerebral haemorrhage (ICH) (red crescent) is shown. Genotype is reported as APP duplication present (dup), absent (-), or not determined (ND). The French kindreds (Wallon *et al.* ^24^) are not shown, as there are insufficient data provided for this purpose. Owing to limitations of the original article, it was not possible to report the genotypes for the Finnish kindred: nine of the affected individuals carry dup-APP, but it is not reported which of the family members this applies to.

The phenotype of dup-APP is one of a highly penetrant AD dementia (frequently associated with seizures ^17, 18, 22^) with an onset age of between 39 and 64 years ^26^. Significantly, carriers of dup-APP suffer a strikingly high rate of ICH. We estimate that this occurs in approximately a third of the published cases ( Table 1 and Figure 1). These haemorrhages are typical of CAA-ICH: multiple and in a lobar distribution. They represent a serious clinical event and are a frequent cause of death in those affected.

**Table 1.** Summary of intracerebral haemorrhage and dup-APP status in the known kindreds.

Country (reference)	Summation of ICH and dup-APP status	ICH percentage (cases/number)
France (Rovelet-Lecrux *et al.* ^17^, 2006)	Five kindreds 14 cases confirmed dup-APP Four cases of ICH in confirmed dup-APP cases ICH in family 229 likely represents a dup-APP case, but genotyping was not done Two unspecified strokes in non-genotyped individuals	28% (4/14)
France (Wallon *et al.* ^24^, 2012)	Seven kindreds 19 affected individuals Cases of ICH in six out of seven kindreds (unspecified total number)	32% (6/19)
The Netherlands (Sleegers *et al.* ^18^, 2006)	One kindred Four cases confirmed dup-APP No confirmed cases of ICH	0% (0/4)
Finland (Remes *et al.* ^76^, 2004; Rovelet- Lecrux *et al.* ^20^, 2007)	One kindred 14 affected cases (nine confirmed dup-APP) Five cases of ICH	36% (5/14)
UK (McNaughton *et al.* ^22^, 2012)	Five probands (confirmed dup-APP) One ICH	20% (1/5)
Japan (Kasuga *et al.* ^21^, 2009)	Two probands One case of ICH (on computed tomography scan of head)	50% (1/2)
Sweden (Thonberg *et al.* ^25^, 2011)	One proband No ICH	0% (0/1)
Spain (Lladó *et al.* ^23^, 2014)	One proband Presented with ICH	100% (1/1)
		Total: 30% (18/61)

The third column gives the most conservative estimate of the proportion of cases, known to harbour duplication (i.e. having three copies) of the amyloid precursor protein region on chromosome 21 (dup-APP), that are also affected by intracerebral haemorrhage (ICH).

Comparison of DS and dup-APP groups shows that the additional copy of the *APP* gene is sufficient to produce both early onset AD and CAA-ICH. However, although haemorrhagic stroke appears to occur in a significant proportion of elderly people with DS (3.3% to 3.8%) ^14^, people with dup-APP are much more profoundly affected; the occurrence rate is approximately 30% (i.e. nearly 10 times higher than in DS). This suggests that triplication of the rest of chromosome 21 may provide partial protection against the pro-haemorrhagic effects of *APP* duplication.

## Pathophysiological insights

Aβ is primarily deposited in the adventitia and media of involved arterioles, and severity of CAA is classified according to spread through the vessel wall: mild CAA is defined as Aβ in the adventitia and some deposits between smooth muscle cells in the media, which are restricted to the tunica media without death of smooth muscle cells. Moderate CAA involves replacement of smooth muscle cells by Aβ and thickening of the media without disruption of the blood-brain barrier (BBB). Severe CAA is defined as extensive Aβ deposition with fragmentation or double-barrelling of the vessel wall, fibrinoid necrosis, and formation of aneurysms ^27^.

The order in which vessels are affected typically follows a particular sequence; the leptomeningeal arteries are the first to show signs of pathology, followed by penetrating arterioles in the neocortical grey matter. Furthermore, vessels in the posterior regions of the brain (such as the occipital lobe) are especially affected, although the frontal cortex has also been named as a relatively early site, followed by vessels of the olfactory cortex, hippocampus, and cerebellum, while deep grey and white matter are usually spared ^3, 28, 29^.

**Figure 2.**
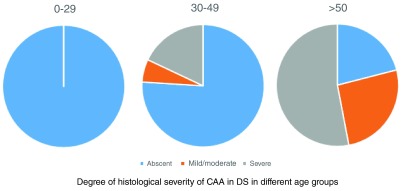
Histological severity of cerebral amyloid angiopathy (system of Vonsattel *et al.* ^27^) seen in post mortem studies of people with Down syndrome in different age groups. Age ranges are indicated above charts. Data are reproduced from 5.

ICH due to CAA is typically lobar, and recurrent or multiple, and may occur in the absence of other risk factors for haemorrhage, such as hypertension. Once haemorrhage has occurred, the result is extensive neuronal death as well as a local immune response from microglia, astrocytes, and other immune cells. The salience of ICH in the phenotype of dup-APP is mirrored by the severity of underlying CAA reported in neuropathology studies. In all 13 cases of dup-APP in which neuropathology has been studied (age range of 48 to 58 years), the histological grade of CAA was moderate to severe and CAA was found in every brain reported ^17, 18, 20, 23^. By contrast, CAA is not a universal finding in people with DS ( Figure 2, data taken from 5). Although most post mortem examinations on people with DS over the age of 50 show CAA from a moderate to severe degree ^5, 30– 32^, a significant proportion (approximately one in five) ( Figure 1) remains completely unaffected by CAA.

These observations suggest a complex relationship between increased APP gene dosage and CAA-ICH; individuals with DS show an increased prevalence of CAA-ICH compared with the euploid population but lower prevalence compared with dup-APP individuals. This suggests some degree of protection, but the mechanisms mediating this relationship are as yet unknown. We will consider the possibilities in the following sections.

## Aβ 40 versus Aβ 42

The 40-amino-acid peptide Aβ (Aβ 40) is more soluble than the longer Aβ 42 peptide. Aβ 40 tends to be the major form of Aβ in the artery walls in CAA, whereas Aβ 42 is more prominent in plaques. The ratio of Aβ 40/42 seems to determine to some extent whether Aβ is deposited in brain parenchyma or in the vessel walls. In mutations where Aβ 42 is the prominent form of Aβ, such as the Indiana and London APP mutations, vascular amyloid seems to be a less prominent feature than parenchymal plaques ^33^. In contrast, a high Aβ 40/42 ratio may promote CAA ^3^, as seen in CAA Dutch type ^34^.

Cellular studies using cortical neurones generated from induced pluripotent stem cells harbouring APP duplication or DS (trisomy of chromosome 21) have addressed the issue of Aβ 40/42 ratio. Both dup-APP ^35, 36^ and trisomy 21 ^37, 38^ cells overproduce Aβ peptide compared with control cells, although the relative overproduction has not been compared in the same study protocol. Surprisingly, in dup-APP cells, the Aβ 40/Aβ 42 ratio is unchanged compared with control cells, which is also the case in mouse studies of overexpression of wild-type *APP*, resulting in an increase of both Aβ 40 and Aβ 42 with the ratio preserved ^34^. In contrast, trisomic cells exhibit an increased Aβ 40/Aβ 42 ratio compared with dup-APP and control cells ^35^.

In post mortem studies, the composition of vascular and parenchymal amyloid appears very similar in dup-APP and DS brains when stained with antibodies specific to either Aβ 40 or Aβ 42. Older individuals with DS ^30, 31^ and dup-APP cases ^17, 19^ have extensive Aβ 40 deposition in vascular walls, preceded temporarily by low-level Aβ 42 deposition in DS ^31^. Parenchymal plaques are present in both groups to a similar extent and stain mostly prominently for Aβ 42.

Given the cellular data, it seems that the Aβ 40/42 ratio may be elevated in DS compared with dup-APP and controls; however, this is not reflected strongly in histopathology studies, which do not suggest significant differences in neuropathology between DS and dup-APP. An increased Aβ 40/42 ratio in DS would predict more severe CAA and predisposition to ICH; however, it does not explain the relative protection compared with dup-APP. Other mechanisms may be at play.

## Aβ clearance in Down syndrome

Increased amyloid in the brain can be the result of either increased production or reduced clearance. As discussed in the preceding section, there is increased production of amyloid in both dup-APP and DS because of the presence of an extra copy of the APP gene in both cases. The Aβ 40/Aβ 42 ratios do not explain the apparent difference between dup-APP and DS in severity of CAA and prevalence of ICH. It is possible that a difference in clearance of vascular amyloid is the key factor.

Aβ is cleared from the brain by several pathways: (1) endocytosis by astrocytes and microglial cells ^39^, (2) enzymatic degradation ^40^, or (3) removal through the BBB ^41^ or along peri-arterial spaces ^42^.

Microglia are the brain’s tissue macrophages; they have been shown to clear Aβ by endocytosis and internal degradation ^39^. However, their exact role in this process is still poorly understood. Recent post mortem studies of brains from individuals with DS who were not older than 40 (i.e. before onset of AD) showed a heightened neuroinflammatory response, which was further increased in older individuals with DS and AD. Microglial cell activation increases with age in DS ^43^ but may be lower compared with sporadic AD cases, despite higher levels of Aβ accumulation, and DS brains were characterised by a unique inflammatory phenotype associated with the formation of immune complexes (M2b) ^44^. The authors hypothesised that accumulation of CAA may result in vascular leakage, with extravasation of IgG into the brain, which in turn may promote the M2b phenotype. Intriguingly, previous work by this group showed that an M2b inflammatory phenotype induced by IgG infusions into brains of an amyloid mouse model promoted clearance of amyloid deposits, suggesting a protective mechanism ^45^. However, if specific changes in neuroinflammation and microglial cells exist in DS, they have not yet been fully investigated and neither has the nature of these mechanisms been explored in dup-APP. Furthermore, since the bulk of extracellular Aβ clearance is via the BBB or interstitial fluid flow ^46^, it seems unlikely to be the main factor accounting for the relative protection against CAA-ICH in DS as compared with dup-APP.

Clearance of Aβ locally is also performed by the cerebrovascular smooth muscle cells and astrocytes through the low-density lipoprotein receptor-related protein-1 (LRP1)-mediated endocytic pathway ^47^. The levels of LRP1 are reduced in patients with AD, and LRP1 levels also decline with age ^48^. Recently, assays were developed to model these processes by using induced pluripotent stem cell models ^49^. The effects of dup-APP and trisomy 21 on these processes are yet to be studied.

Physiological degradation of Aβ involves metallopeptidases such as neprilysin (NEP) ^46^. NEP degradation of Aβ seems to be protective against CAA ^50^. NEP expression in vascular smooth muscle cells is inversely correlated with degree of vascular Aβ ^51, 52^, and a polymorphism in the NEP promotor region that may reduce NEP transcription levels is associated with more severe CAA ^53^. There is little published research on enzymatic degradation of Aβ in DS. However, one study by Miners *et al.* (2010) showed NEP levels to be increased in DS brains (age range of 10 to 80 years) compared with non-DS controls, and NEP level was strongly correlated with insoluble Aβ concentration ^54^. This contrasts with evidence from sporadic AD showing decreased NEP immunoreactivity compared with age-matched controls ^55^. It is possible that in DS there is greater capacity for NEP expression conferring some protective effect against CAA.

Transport of Aβ across the BBB is receptor mediated. The low-density lipoprotein pathway transports Aβ from the brain interstitial and cerebrospinal fluid compartments into the circulation ^56^. To the best of our knowledge, specific abnormalities of the BBB have not been demonstrated in DS; however, lipid processing is known to be abnormal ^57^, indirectly supporting the idea that BBB-mediated efflux of Aβ from the brain could be altered in this group.

A proportion of neuronally produced Aβ flows with the interstitial fluid along perivascular spaces to be excreted into the cerebrospinal fluid and drained into the systemic circulation ^42^. Although the contribution of perivascular drainage to CAA is by no means clear, it has been proposed to be a compensatory mechanism when other routes fail, and this may underlie the strong association between age and the development of CAA and AD pathology in the general population. Perivascular drainage is proposed to rely on counter-current flow of lymphatics driven by the arterial pulsation ^58^. As the arteries are increasingly affected by atherosclerosis or inflammation during ageing, they become more rigid with less effective contractile function and perivascular drainage. As individuals with DS appear to be somewhat protected against atherosclerosis ^57^, this could be another protective mechanism, resulting in better Aβ drainage and less severe CAA.

## Other potentially protective mechanisms

DS may be associated with several other protective mechanisms. It is possible that DS differs from dup-APP in the response to Aβ-related cell damage. Evidence from mouse model studies shows that immunotherapy against amyloid increases CAA and may also result in increased micro-haemorrhages ^59^, suggesting an important role for the immune system in the pathophysiology of CAA. The innate immune system may differ in DS, thus affecting response to AB deposition. This possibility remains to be explored. Furthermore, the effect of free radicals may contribute to vascular damage, and although oxidative stress is a prominent feature of DS, it has been shown that increased activity of some anti-oxidant enzymes such as superoxide dismutase (SOD1, encoded on chromosome 21 and triplicated in DS) is associated with less cognitive decline, suggesting another potential protective mechanism ^60, 61^. In contrast, experiments using the Tg2576 mouse model have resulted in the suggestion that Aβ-induced oxidative stress causes DNA damage and excess opening of TRPM2 calcium channels, leading to calcium overload, which in turn results in endothelial dysfunction ^62^.

Specific apolipoprotein E ( *APOE*) genotypes (ε4 and ε2) are known risk factors for more severe CAA and ICH in the general population ^63– 65^. Two of the seven cases of ICH in DS report APOE genotype, both carrying high-risk variants (ε2/ε4 ^7^ and ε4/ε4 ^9^). This contrasts with dup-APP, where all 11 cases of ICH in which APOE genotype is reported carry a low-risk variant (ε3/ε3 n = 9, ε3/ε4 n = 2). These very limited data suggest an importance of *APOE* genotype as a risk factor for CAA-ICH in DS and possible *APOE* independence of ICH in dup-APP; further investigation is needed. However, it is noteworthy that *APOE* is mapped to chromosome 19, not chromosome 21, and this by itself is unlikely to explain differences between DS and dup-APP groups. In this regard, the ATP binding-cassette G1 ( *ABCG1*) gene may be a more relevant candidate, as it is located on chromosome 21 and is thought to be responsible for cholesterol efflux onto apolipoproteins ^66^. However, cellular studies provide conflicting evidence suggesting that *ABCG1* overexpression may increase ^67^ or reduce ^68^ Aβ production. Evidence from mouse models is also conflicting. One study examined transgenic mice with a sixfold overexpression of *ABCG1* that did not exhibit increased levels of Aβ ^69^; by contrast, *APOE ε4* mice treated with bexarotene, an agent that indirectly upregulates *ABCG1* and *ABCA1*, reversed hippocampal Aβ 42 deposition ^70^. The evidence is unclear and its relation to CAA and ICH even more so.

Finally, individuals with DS of all ages are less at risk from hypertension than their peers in the general population (incidence rate ratio 0.3, 95% CI 0.3 to 0.4) ^71^. Although hypertension has not (yet) been clearly related to CAA-ICH in DS and in fact gives rise to a different pattern of haemorrhage, it is theoretically possible that higher blood pressure may increase the likelihood of aneurism and bleeds in vessels severely affected by CAA in those with dup-APP compared with individuals with DS.

## Further research

Further epidemiological data are needed in DS regarding haemorrhagic stroke—a diagnostic category, not a single entity. CAA-ICH can be distinguished from other forms of haemorrhagic stroke on clinical grounds by using the validated Boston criteria ^4^. The age-related risk for CAA-ICH in DS should be explored using susceptibility weighted imaging magnetic resonance imaging scans to detect microbleeds, which will allow comparison against dup-APP and sporadic AD to confirm relative burden of disease in these groups.

The role of factors involved in the clearance of Aβ in DS should be explored in more depth, as this could help to reveal potential drug targets to reduce CAA and associated ICH. Specifically, we have identified gaps in knowledge of the relationship between Aβ 40/Aβ 42 ratios and development of CAA and ICH in DS, on one hand, and Aβ clearance by endocytosis, enzymatic degradation, and removal through the BBB, on the other. Furthermore, direct comparison between DS and dup-APP cases and models is required. In this regard, mouse modelling with partial triplication of areas of chromosome 21 might identify an area of the chromosome that modulates the risk of CAA-ICH. CAA develops in several AD mouse models, including the Tg2576 ( *APP* expressed under the PrP promoter) ^72^, and J20 mouse model ( *APP* transgenics with Swedish and Indiana mutations), particularly after 11 to 12 months of age ^73, 74^. The Tg-SwDI mouse is the most widely used model for studying CAA, containing the Swedish, Dutch, and Iowa mutations and developing CAA at 6 months ^75^. Therefore, different partial trisomy strains could be crossed to transgenic mice expressing forms of APP that give rise to CAA and micro-haemorrhages; double mutant progeny could be assessed for CAA to see whether regions of chromosome 21 mediate increased or reduced pathology compared with mice carrying the APP transgene alone. If such a region were found, then it would give us dosage-sensitive candidate genes affecting the risk of CAA-ICH. Table 2 summarises some of the important unanswered questions generated by this review.

**Table 2.** Summary of further research questions resulting from this review and corresponding suggestions for further enquiry.

Further research questions	Possible investigative strategy
What is the true age-related prevalence of CAA-ICH in individuals with DS?	1. Further epidemiological studies comparing DS against general AD population and dup-APP are required 2. Susceptibility weighted imaging magnetic resonance imaging studies to detect microbleeds in DS population
Is there a region of chromosome 21 that specifically modifies the risk of CAA?	Different partial trisomy mouse strains could be crossed with transgenic mice expressing forms of *APP* that give rise to CAA; double mutant progeny could be assessed for CAA and micro-haemorrhage
Does clearance of Aβ in DS differ from dup-APP?	1. Establish the relationship between Aβ 40/Aβ 42 ratios and development of CAA and ICH in DS by using neuropathological studies 2. Experimental studies of Aβ clearance by endocytosis, enzymatic degradation, and removal through the blood-brain barrier by using animal and cellular models 3. Compare DS and dup-APP by using neuropathological and cellular studies and animal models
What is the role of APOE genotype in CAA-ICH in the DS population and in dup-APP?	Population-based cohort study in DS individuals stratified by *APOE* genotype with imaging-confirmed micro-haemorrhage or ICH as the main outcome measure; collect similar data in families with dup-APP
To what extent do other factors influence CAA development in DS?	1. Explore the relationship between the immune system and CAA-ICH by using genomic, neuropathological, and immunological studies 2. Explore the role of oxidative stress, and in particular antioxidant enzymes such as SOD, in CAA-ICH using mouse model experiments and longitudinal human biomarker studies 3. Explore the role of vascular risk factors such as blood pressure in ICH in DS by using population-based epidemiological studies

Aβ, amyloid-beta; AD, Alzheimer’s disease; *APOE*, apolipoprotein E; *APP*, amyloid precursor protein; CAA, cerebral amyloid angiopathy; DS, Down syndrome; dup-APP, duplication (i.e. having three copies) of the amyloid precursor protein region on chromosome 21; ICH, intracerebral haemorrhage; SOD, superoxide dismutase.

## Conclusions

There is much variation between individuals with DS and development of clinical dementia and associated CAA and ICH. Variability in phenotypic and pathological expression, however, is not unique to DS but has also been reported in familial AD, suggesting the presence of genetic and non-genetic factors with disease-modifying effects. Intriguingly, individuals with DS appear to have much lower risk for developing ICH as well as some indications of less severe CAA when compared with families with dup-APP, despite also having three copies of the *APP* gene, suggesting that other genes on chromosome 21 may provide some protection against the effects of *APP* overdose. This review of the literature suggests that this lower prevalence and seemingly protective effect of trisomy 21 may be related to a difference in the clearance of Aβ, although other factors such as neuroinflammation, atherosclerosis, oxidative stress, and lower blood pressure could also have a role. Insights into these factors may provide important information about mechanisms of disease, which can be exploited to identify treatment strategies. For example, if it turns out that low blood pressure helps to protect individuals with DS from ICH, then that would suggest an important strategy to offer individuals from families with familial AD mutations at risk for CAA and ICH.
